# Evaluating a Novel Visualization Device for Improving File Insertion Accuracy During Root Canal Treatment

**DOI:** 10.1055/s-0045-1806961

**Published:** 2025-05-01

**Authors:** Abubaker Qutieshat, Rajmohan Sivamani Chidambaram, Gurdeep Singh, Samiya Al Ghammari, Ritaj Al Busaidi, Iman Al Sukaiti, Fatima Al Rawas, Mariam Al Balushi, Zahra Al Lawati, Doaa Ahmed, Taif Al Shirawi

**Affiliations:** 1 Restorative Dentistry, Oman Dental College, Muscat, Oman; 2 Restorative Dentistry, College of Dental Medicine, University of Sharjah, Sharjah, United Arab Emirates

**Keywords:** posture, root canal, visual bias, perception

## Abstract

**Objectives:**

This study aimed to evaluate whether adopting a horizontal viewpoint, facilitated by a novel digital assistive device, could enhance endodontic file placement accuracy and reduce operator-dependent variability during root canal treatment.

**Materials and Methods:**

A total of 40 modified upper jaw dental stone models, each accommodating a plastic tooth replacing the upper right second molar, were divided into two groups ( *n* = 20 each). The mesiobuccal canal of each tooth was prepared to a standardized working length of 21.0 mm and a working width of size 35. A size 40 file with a stopper preadjusted to 19.0 mm was then inserted. In the first (conventional) group, files were placed without assistance; in the second (device-assisted) group, a horizontal-view digital device was designed, developed, and used to align and insert the file. The second operator, blinded to the device's purpose, performed all insertions under simulated clinical conditions. An intraoral scanner subsequently captured the vertical distance from the stopper's bottom surface to a standardized anatomical landmark. Pairwise comparisons between the two groups were computed using alignment software to account for potential measurement artifacts.

**Statistical Analysis:**

Normality in both groups was confirmed via the Shapiro–Wilk test. An independent-samples *t* -test compared mean vertical distances. Additionally, differences in stopper positioning were calculated for all aligned virtual models in CloudCompare.

**Results:**

The device-assisted group exhibited a significantly shorter mean stopper-to-landmark distance (0.425 mm, standard deviation [SD] = 0.225) than the conventional group (0.971 mm, SD = 0.432) ( *t* = −5.014, *p* = 2.534 × 10 ^−5^ ). Pairwise analysis highlighted closer apical positioning in the device-assisted group, closely matching pilot study findings that a 26.57° vertical viewing angle can distort perceptions by 0.5 mm. The device's mean intraoral mounting time was 224 s (SD = 35.2), considered negligible over the full treatment duration.

**Conclusion:**

Adopting a horizontal perspective with a novel digital assistive device significantly improved file placement accuracy and reduced operator-dependent variability during root canal treatment. Optimizing this device's design and assessing its cost-effectiveness may facilitate broader clinical adoption and further enhance endodontic procedural outcomes.

## Introduction

Precision in endodontic procedures is critical for achieving successful treatment outcomes. A fundamental aspect of such precision is accurately determining and maintaining the working length of root canals, defined as the distance between the apical limit of instrumentation and the coronal reference point. Proper length determination ensures that instruments remain confined within the canal, preventing injury to the periapical tissues and facilitating precise obturation of the root canal system. 1 Conversely, inaccuracies can result in incomplete instrumentation, underfilling, and procedural mishaps, leading to persistent pain, periapical tissue damage, and an increased incidence of treatment failure. 2

Errors in working length determination, such as losing the reference point or facing challenges in confirming cone fit, are a predominant source of complications in clinical settings. 3 4 5 These challenges highlight the critical need for strategies and tools that enhance precision and reduce operator-dependent variability, both in endodontic education and clinical practice.

Traditional methods for adjusting and maintaining the stopper position on endodontic files heavily rely on the operator's visual acuity and manual dexterity. Typically, the reference point on the tooth and the stopper's landing are observed from a vertical perspective, inherently limiting the operator's ability to assess precise contact. Verifying the stopper's position from a horizontal perspective often requires operators to adopt awkward postures or unsustainable viewing angles, which introduces significant variability. These postures also place considerable strain on the operator's neck and back, compounding the challenges of maintaining precision. 6 7 8 Implementing assistive devices that provide a stable horizontal perspective could significantly improve stopper placement accuracy while alleviating these ergonomic risks.

Advancements in digital technology have paved the way for assistive devices designed to enhance accuracy and consistency in clinical workflows. A pioneering study by Song et al explored the use of augmented reality in guided endodontic procedures, emphasizing improved spatial accuracy while maintaining a focus on the procedural field. 9 Building on this progress, the present study shifts focus to maintaining working length and evaluates a novel device designed to improve the precision of file placement during endodontic procedures by mitigating the visual biases inherent in conventional methods.

This study therefore seeks to determine whether a horizontal perspective, coupled with a novel digital assistive device, can enhance the accuracy and reduce variability in stopper placement. In doing so, it addresses an important gap in endodontic practice and holds potential for improving procedural outcomes and long-term treatment success.

## Materials and Methods

This bench-top experimental study involved both the design and construction of an assistive technology device and testing on synthetic plastic models. No human participants or animal subjects were used. The research protocol was reviewed and approved by the Institutional Review Board in accordance with institutional guidelines for nonbiological experimental investigations.

A total of 40 modified upper jaw dental stone models were utilized in this study. Each model was prepared with a slot filled with wax to accommodate an anatomical plastic tooth (Tooth #27, Anatomical Pulp Model B1X Series, IRTS) replacing the upper right second molar.

To standardize the experimental setup, two different operators were involved in the study. The first operator accessed the teeth and manually prepared the mesiobuccal canal of each tooth to a size 35 with a working length of 21.0 mm. This operator's role was limited to canal preparation, ensuring that the procedural steps leading up to file insertion were uniformly executed. After the teeth were prepared, the models were divided into two groups of 20 models each, forming the basis for subsequent file insertions and assessments by the second operator. Both operators were highly qualified and experienced clinicians with postgraduate training in endodontics.

Before the second operator began, the stopper was glued to a size 40 file and adjusted to a working length of 19.0 mm using a digital microscope equipped with a graduated ruler (Celestron MicroDirect 1080p HDMI, Torrance, California, United States). By setting the length at 19.0 mm, the prepared canal width of approximately 0.39 mm at that length closely matched the file's 0.40 mm tip diameter, ensuring file engagement. This adjustment also allowed for potential placement errors along the long axis, whether positive or negative.

In the first group, the second operator inserted the preadjusted file into the mesiobuccal canal of each tooth without any assistive device. These insertions were performed under simulated clinical conditions, with the operator seated appropriately against a phantom head to replicate real-world scenarios. In the second group, the same process was undertaken using a horizontal-view device designed to enhance accuracy and ease of file placement to length.

To maintain procedural validity and eliminate bias, the second operator was blinded to both the specific purpose of the horizontal-view device and the existence of two distinct experimental groups. This approach ensured that any observed differences in stopper placement accuracy could be directly attributed to the horizontal-view system rather than operator proficiency or other factors. Moreover, the second operator did not know the original working length or the final canal preparation size, receiving only instructions to use a size 40 file at a 19 mm length.

**Fig. 1:**
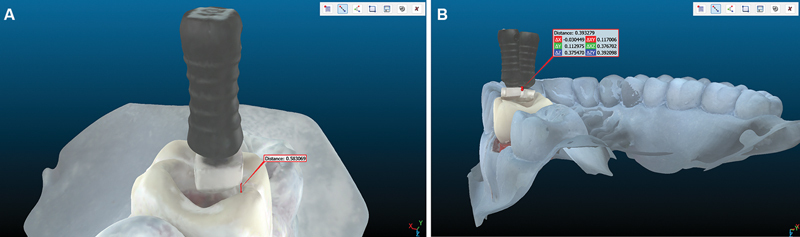
( **A** ) CloudCompare-based measurement of the vertical distance between the stopper's bottom surface and the juncture of the mesiobuccal and distobuccal cusps on the buccal aspect. ( **B** ) Pairwise alignment of virtual models in CloudCompare enabling a systematic comparison of stopper positioning between the device-assisted and nonassisted groups.

Following file insertions in both groups, the models were digitized using a Dentsply Sirona Primescan intraoral scanner. CloudCompare software (version 2.13.1) first served to measure the vertical distance between the file stopper's bottom surface and the lowest point at the juncture of the mesiobuccal and distobuccal cusps ( Fig. 1a ). 10 11 12 Notably, this juncture was not the actual reference point used during tooth preparation or file insertion, but rather a standardized landmark selected for CloudCompare analysis only, because a perfectly vertical line could be drawn from that point to the stopper's bottom surface. CloudCompare was then employed to align and compare all digital models from the device-assisted group against every model from the nonassisted group, enabling the calculation of pairwise differences in these same vertical distances for each model pair ( Fig. 1b ). Subsequently, the differences in vertical distances were calculated for each pair of aligned models by subtracting the measured distance of the conventional group's stopper from that of the device-assisted group. These differences represent the deviation between the two aligned stopper positions relative to the reference point on the tooth surface. Negative values were retained as recorded, indicating cases where the nonassisted stopper was positioned closer to the reference point compared to the device-assisted stopper.

### Determination of Viewing Angle

Before initiating the main study, a pilot study was performed to quantify the operator's viewing angle ( *θ* ) and distance ( *θ* ) during simulated file insertion. A total of 30 participants wore goggles and positioned themselves as if placing a file into a tooth. A thread connected the goggles' center to the specific point on the tooth where each participant-focused. Using a protractor, the angle formed between the thread and a vertical line was recorded for each individual, while the thread's length provided a measure of the operator's distance ( *L* ) from the operative field. The operator's distance ( *L* ) was measured to approximate a realistic working distance in clinical scenarios and to contextualize the theoretical calculation of the effective vertical displacement.

These measurements were then applied to calculate the theoretical effective vertical displacement ( *d*_effective_ ) based on trigonometric relationships.





A vertical displacement of 1 mm was assumed (the smallest practical distance) to represent the practical precision limit of an endodontic ruler. Substituting 1 mm into the equations gave:



This pilot study aimed to illustrate the magnitude of potential perceptual error resulting from an oblique viewing angle. The theoretical value derived from these trigonometric calculations was then compared with the measured difference between the device-assisted and nonassisted approaches, confirming whether the measured offset aligns with the pilot study's predictions and emphasizing how a horizontal perspective can mitigate viewing-angle bias.

### Creation and Setup of the Horizontal-View Device (CuspTracker)

The horizontal-view (i.e., CuspTracker) device was built around a Raspberry Pi 4 Model B, a Raspberry Pi Camera Module 2 (8 Megapixel, 1080p, Sony IMX219 sensor), an HDMI cable, a 22-inch HD monitor, and an AC power source. The Raspberry Pi 4 was configured with the Raspberry Pi OS installed on a microSD card. Once the operating system was loaded, the camera module was enabled in the Pi's configuration settings. The system was programmed to provide real-time, high-definition video streaming of the operative field using a Python-based setup that utilizes Flask, Picamera, and OpenCV libraries. 13 The code initializes a Flask web server running on the Raspberry Pi, captures 1080p video frames at 30 frames per second, and streams these frames to a web interface. When executed, the code provides a continuous stream of JPEG-encoded frames at a localhost IP address.

**Fig. 2:**
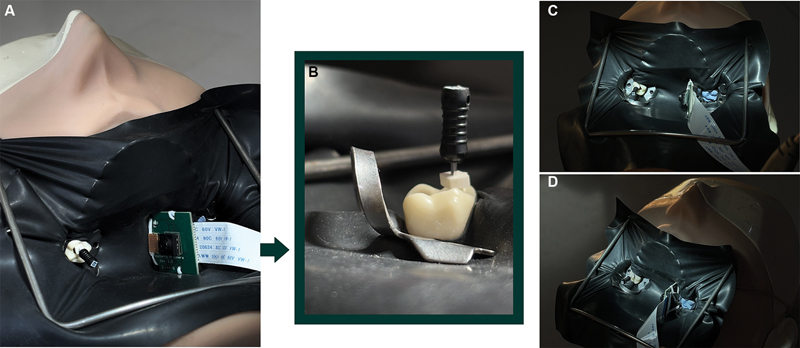
( **A** ) Assembly showing the device positioned without impeding treatment. ( **B** ) Real-time image stream displayed on the monitor. ( **C** ) Assembly with the contralateral tooth clamped for camera placement. ( **D** ) Camera sensor secured to the clamp using Teflon tape.

Once the camera system was assembled and configured, the rubber dam was placed to isolate the treated tooth using a conventional clamp. To affix the horizontal-view device securely, a second clamp was positioned on the contralateral tooth, providing stability for the camera assembly while positioning the lens horizontally toward the treated tooth ( Fig. 2 ). The high-definition, real-time video feed provided a horizontal view and was displayed on the monitor. The time required to mount the horizontal-view device intraorally, including aligning the device and securing it to the contralateral clamp, was recorded.

## Results

The pilot study included 30 participants. The median viewing angle ( *θ* ) was determined to be 26.57° with the vertical line (standard deviation [SD] = 5.2), equivalent to 63.43° with the horizontal line. The operator's distance ( *L* ) was measured as 300 mm (SD = 33.4).

**Fig. 3:**
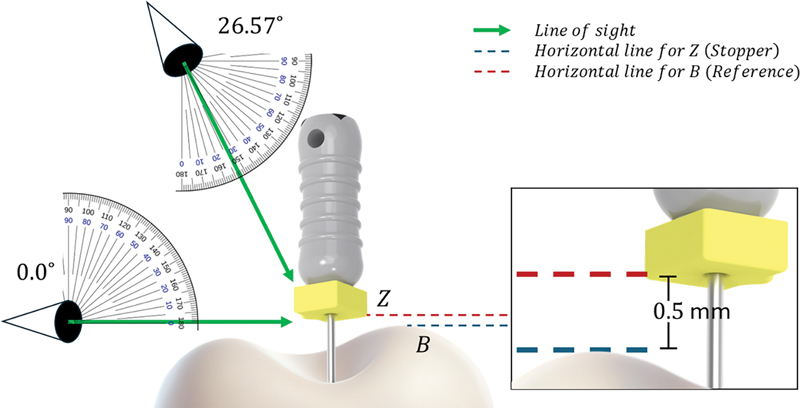
The effect of the operator's viewing angle (26.57°) on the perception of Z (stopper bottom edge) and B (reference point). At 26.57°, the line of sight creates an illusion where Z and B appear to touch, despite a vertical displacement of approximately 0.5 mm. This highlights the perceptual bias introduced by oblique viewing angles in close-up dental procedures.

Using these values, the effective vertical displacement ( *d*_effective_ ) was calculated as:



This calculation indicates that, for an actual vertical displacement of 1 mm, the operator might perceive a displacement of approximately 0.5 mm due to the viewing angle ( Fig. 3 ).

The study evaluated 40 dental models, split evenly between the device-assisted and conventional groups (20 models each). Table 1 summarizes the mean distances between the bottom of the file stopper and the lowest point at the juncture of the mesiobuccal and distobuccal cusps on the buccal aspect for each group. The mean distance in the device-assisted group was 0.425 mm (SD = 0.225), significantly shorter than the conventional group's mean of 0.971 mm (SD = 0.432). The mean difference of −0.546 mm indicates that, on average, the stopper in the device-assisted group was more apically positioned than in the conventional group.

**Table 1:** Measured distance between the bottom of the stopper and the lowest point at the juncture of the mesiobuccal and distobuccal cusps on the buccal aspect

Group	Mean (mm)	SD (mm)
Device-assisted	0.425	0.225
Conventional	0.971	0.432
**Difference**	−0.546	

Abbreviation: SD, standard deviation.

Normality tests were conducted to validate the use of parametric analysis. The Shapiro–Wilk test confirmed normal data distribution for both groups (device-assisted: *W* = 0.945, *p* = 0.280; conventional: *W* = 0.914, *p* = 0.100). An independent-samples *t* -test demonstrated a statistically significant difference between the two groups ( *t* = −5.014, *p* = 2.534 × 10 ^−5^ ), indicating that the difference in stopper placement precision was highly significant ( *p* < 0.05).

**Fig. 4:**
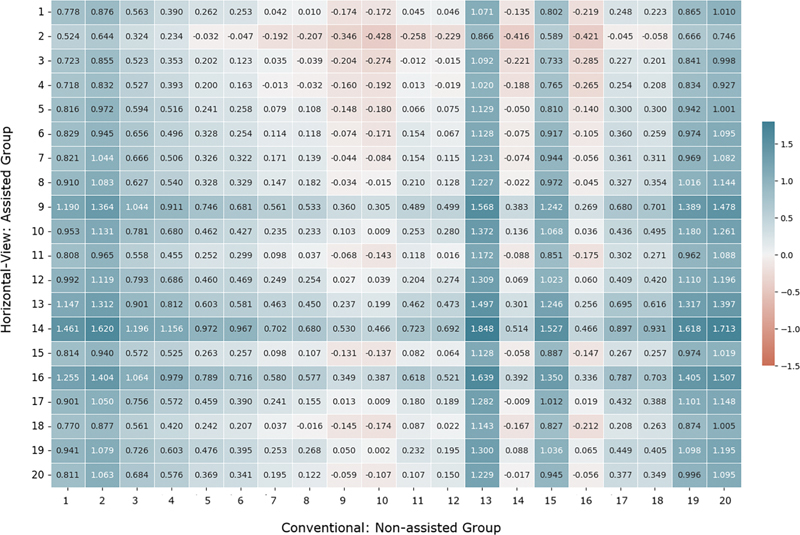
Heatmap of differences in stopper positions between device-assisted and conventional groups. Each cell represents the vertical distance difference (in mm) between one device-assisted model (rows) and one conventional model (columns).

To further explore the differences, comparisons between all models in the device-assisted and conventional groups were conducted. The results are visualized in Fig. 4 , which presents a heatmap of the differences between stopper positions across all combinations of models. Blue areas in the heatmap indicate positive differences (greater apical positioning in the device-assisted group), while red areas indicate negative differences (greater apical positioning in the nonassisted group). White cells represent negligible or no differences. The color intensity reflects the magnitude of the difference.

The device-assisted group consistently demonstrated more apical stopper positioning compared to the conventional group, as reflected by the heatmap and statistical analysis. The visualization highlighted the stability and reproducibility of the device-assisted group's results, with the majority of differences skewing toward improved precision for the device-assisted group.

During the evaluation, the mean time required to mount the visualization device intraorally was recorded. The mounting process, including aligning the device and securing it to the contralateral clamp, took an average of 224 seconds (SD = 35.2).

## Discussion

The findings of this study indicate a notable alignment between the theoretical displacement calculated in the pilot study and the observed differences in the main study. In the pilot study, an effective vertical displacement of 0.5 mm was derived based on a viewing angle of 26.57° with the vertical axis and an operator distance of 300 mm. This calculation predicted the perceptual distortion introduced by the operator's line of sight. Remarkably, the mean difference in stopper placement observed in the main study also approximated 0.5 mm, further validating the theoretical framework and reinforcing the role of viewing angles in shaping operator perception during endodontic procedures.

The prototype device evaluated in this work shows significant potential for maintaining working length during the cleaning and shaping phase of root canal treatment, provided it is mounted intraorally in the proper position. The marked discrepancy in working length without the device highlights its ability to deliver the precision required for successful treatment. By providing a horizontal perspective, the device minimized the influence of the operator's vertical viewing angle, enabling more consistent and accurate stopper placement.

These findings build on previous research underscoring the importance of operator perspective and posture in achieving precise endodontic outcomes. 6 7 Studies on ergonomics have shown that visual bias and awkward viewing angles can compromise accuracy and contribute to musculoskeletal strain among dental practitioners. 8 By offering a horizontal viewpoint, the prototype helps minimize the effects of vertical viewing angles, supporting a more consistent and accurate flow of root canal treatment.

These results also align with broader initiatives employing high-definition digital imagery and computer vision for real-time intraoperative assistance, where benefits include tool detection, identification, and pose tracking during operative stages. 14 Much like minimally invasive surgical procedures that capitalize on endoscopic video feeds, integrating real-time capture and processing can significantly enhance operator–field synergy. 15 Transitioning from purely analog visualization to dynamic digital feedback reduces operator-dependent variability and can improve both efficiency and accuracy within the clinical workflow.

Unlike augmented reality systems relying on head-mounted displays, 9 which may disrupt the operator's focus on the procedural field, the monitor-based solution described here is designed to optimize ergonomics and seamlessly integrate into clinical workflows. During testing, the device required a mean time of 224 seconds to mount, which is a significant increase compared to the brief period needed to apply a conventional rubber dam. However, this additional time is negligible over the full duration of a root canal treatment session. Further refinements to the design and functionality could streamline mounting and improve efficiency. Notably, the horizontal view device is cost-effective, with total component costs estimated at approximately $200. This affordability makes it a viable option for clinical practices seeking to enhance precision without incurring the high expenses associated with advanced imaging technologies. By reducing procedural errors and the need for retreatments, the device could offer long-term cost savings in clinical settings.

The methodology employed in this study was designed to evaluate the device's effectiveness in minimizing visual bias during clinical procedures. A key component of this approach was the use of standardized measurement techniques to address potential inconsistencies when analyzing scanned models, particularly in defining the anatomical landmark on the tooth. Ensuring a uniform method for identifying and measuring specific points on the scanned models was critical to validating the findings. The incorporation of a model alignment methodology further accounted for potential measurement errors, allowing for accurate comparisons between groups. 16 This systematic approach minimized artifacts and ensured that the observed differences in stopper placement were attributable to the intervention rather than measurement inconsistencies. As a result, the study provides a robust foundation for analyzing the device's ability to enhance procedural precision.

While this study demonstrated the device's effectiveness using standardized plastic teeth and dental stone models, it is important to recognize potential differences when applying these findings to real clinical scenarios. Synthetic models offer uniformity and ease of manipulation, but they lack the anatomical variability and complexity of natural teeth and surrounding biological tissues. Additionally, the device's mounting process, while straightforward, introduced extra procedural time which could influence workflow efficiency in a clinical setting. The study design focused exclusively on measuring stopper placement accuracy and did not evaluate the device's broader impact on treatment outcomes or operator ergonomics during prolonged use. Moreover, variability among multiple operators was not assessed, which limits the generalizability of the findings to broader clinical applications. Potential sources of bias include the operator's adaptation to the device over time, which may influence performance in the device-assisted group. Although efforts were made to minimize bias by blinding the operator to the device's specific purpose, familiarity with the experimental setup could have inadvertently affected results. However, if such an effect occurred, it would also highlight an additional benefit of the device, as its intuitive design and ease of integration into clinical workflows could further support its adoption in practice. Future investigations should address these aspects and further explore how the use of this device influences long-term clinical efficacy, such as root canal treatment success rates. This will provide a more comprehensive evaluation of the device's utility in both procedural and outcome-based contexts.

## Conclusion

This work demonstrates a strong capacity to mitigate operator-dependent variability by addressing the perceptual and ergonomic limitations associated with vertical viewing angles. Our results suggest that adopting a horizontal perspective and incorporating real-time digital feedback can effectively maintain the stopper in closer proximity to the reference point on the tooth surface, enhancing procedural precision. The close alignment between the theoretical and observed findings validates the device's conceptual foundation, while the promising results from its initial testing highlight its practical utility. Future research should focus on optimizing the device's design, assessing its cost-effectiveness, and exploring its broader integration into clinical practice.
